# Nanospheres of doxorubicin as cross-linkers for a supramolecular hydrogelation

**DOI:** 10.1038/srep08764

**Published:** 2015-03-05

**Authors:** Qiang Xue, He Ren, Chao Xu, Gang Wang, Chunhua Ren, Jihui Hao, Dan Ding

**Affiliations:** 1 National Clinical Research Center for Cancer, Key Laboratory of Cancer Prevention and Therapy, Department of Pancreatic Cancer, Tianjin Medical University Cancer Institute and Hospital, Tianjin, P. R. China; 2 State Key Laboratory of Medicinal Chemical Biology, Key Laboratory of Bioactive Materials, Ministry of Education, College of Life Sciences, Nankai University, Tianjin. 300071, P. R. China

## Abstract

In this study, we synthesized a peptide of Nap-GFFYGRGD, which could self-assemble into supramolecular nanofibers. The peptide itself could only form nanofibers but not hydrogels due to the relative weak inter-fiber interactions. The resulting nanofibers were then utilized as the vehicles for anticancer drug doxorubicin. It was found that the nanofibers of Nap-GFFYGRGD could not encapsulate doxorubicin, whereas the drug formed nanospheres, which were located at the surface of the nanofibers. Due to the electrostatic interactions between the negatively charged nanofibers and the positively charged doxorubicin nanospheres, the doxorubicin nanospheres were able to serve as a cross-linker to increase the inter-fiber interactions, leading to the formation of stable three-dimentional fiber networks and hydrogels. The resulting doxorubicin-peptide hydrogels were capable of releasing the drug in a sustained manner, which also showed comparable cytotoxicity as compared to free doxorubicin against a variety of cancer cell lines including HeLa and MCF-7 cancer cells. Therefore, this successful example using drug as the peptide nanofiber cross-linkers provided a new strategy for fabricating supramolecular hydrogelation for controlled delivery of anticancer drugs.

Supramolecular hydrogels are formed by the self-assembly of small molecules (hydrogelators) that are biocompatible and easily degradable^1^,^2^,^3^,^4^,^5^,^6^. They are promising soft materials for three dimensional (3D) cell culture^7^,^8^,^9^, controlled delivery of therapeutic agents and proteins^10^,^11^,^12^,^13^,^14^, and regenerative medicine^15^,^16^,^17^,^18^,^19^. In order to trigger the self-assembly of small molecules and the formation of supramolecular hydrogels, external stimuli are required including pH^20^,^21^,^22^, temperature^23^,^24^, and ionic strength changes^25^,^26^,^27^, light irradiation^28^,^29^,^30^, redox^31^, enzymatic and chemical reactions^32^,^33^,^34^, etc. These stimuli can trigger the formation of supramolecular nanostructures (in most cases, nanofibers) of small molecules. The nanostructures further entangle with each other to form 3D networks that would efficiently immobilize water molecules, leading to supramolecular hydrogelations. Recently, the application of supramolecular hydrogels in the delivery of hydrophobic therapeutic agents attracts extensive research interest^10^,^35^,^36^,^37^,^38^,^39^,^40^. The hydrophobic therapeutic agents are able to be physically entrapped in the cavity of 3D fiber networks of hydrogels or in the hydrophobic domain of nanofibers^41^,^42^,^43^. Therapeutic agents can also be developed into hydrogelators to form nanofibers as self-delivery systems^44^,^45^,^46^. In this study, we find that doxorubicin can form nanospheres at the surface of supramolecular nanofibers of a peptide. The formed nanospheres enhance the inter-fiber interaction of the peptide, resulting in supramolecular hydrogelations.

## Results

### Preparation and characterization of doxorubicin-peptide hydrogels

**Figure 1:**
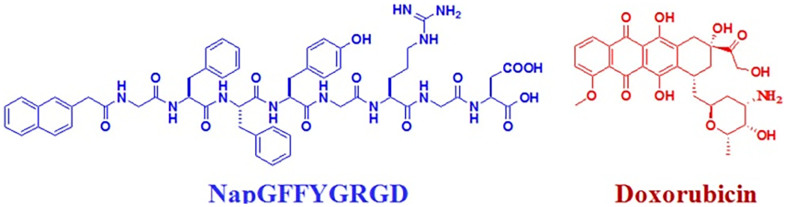
The compounds utilized in this study. Chemical structures of the peptide (Nap-GFFYGRGD) and doxorubicin used for hydrogelations.

The doxorubicin (trade name Adriamycin) is widely utilized for cancer chemotherapy. Nanocarriers have been used to increase its water solubility and reduce its side effects to the heart, such as pegylated liposomal form (trade name Doxil). We opted to use supramolecular nanofibers of peptides to deliver doxorubicin and we chose the peptide of Nap-GFFYGRGD (Fig. 1), because the peptide could self-assemble into nanofibers with good water solubility. In this work, Nap-GFFYGRGD was synthesized by standard solid-phase 9-fluorenyl-methoxycarbonyl (Fmoc) peptide chemistry, and its purity and identity were characterized by ^1^H NMR and LC-MS (Supplementary Fig. S1 and S2). The peptide Nap-GFFYGRGD was firstly reported by Liu and co-workers. It could form nanofiber dispersions but not hydrogels, and it had been used to deliver hydrophobic drugs of curcumin and 10-hydroxy camptothecin (HCPT)^47^. During the experiments, we occasionally found that the addition of doxorubicin led to a sol-gel phase transition, which was different to Liu's results. We therefore conducted further experiments to understand the gelation mechanism.

**Figure 2:**
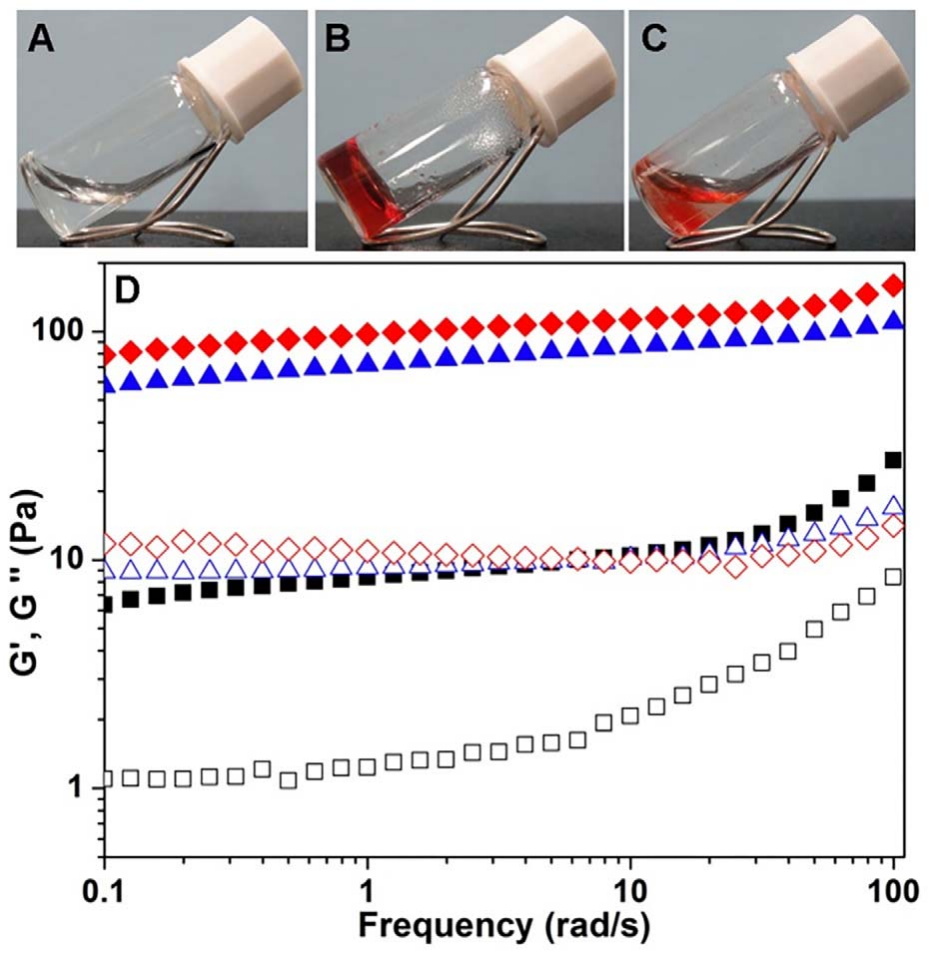
Formation of doxorubicin-peptide hydrogels. Optical images of (A) the peptide in PBS solution at the concentration of 0.5 wt% and pH value of 7.4, and (B) the gel formed by adding 0.1 equiv. of doxorubicin to the peptide solution, and (C) the precipitation formed by adding 0.1 equiv. of doxorubicin to the PBS solution in the absence of the peptide. (D) rheological measurement with the mode of dynamic frequency sweep at the strain of 0.5% of the gels formed by adding different amounts of doxorubicin to the peptide solution (diamonds: 0.3 equiv., triangles: 0.2 equiv., squares: 0.1 equiv., filled symbols: G' and open symbols: G″).

As shown in Fig. 2A, the peptide itself could form a transparent solution in phosphate buffer saline (PBS, pH = 7.4) at the concentration of 0.5 wt% (5 mg/mL), which was consistent with Liu's results. Upon addition of 0.1 equiv. of doxorubicin (0.025 wt%) to the peptide solution, a homogeneous mixed solution was obtained by heating and hydrogels was formed within 5 minutes (Fig. 2B). In the absence of the peptide, doxorubicin would form precipitation in the PBS solution (Fig. 2C), suggesting that the peptide could improve the water solubility of doxorubicin in neutral conditions. The minimum amount of doxorubicin to induce the hydrogelation of the peptide solution (0.5 wt%) was about 0.06 equiv., and up to 0.55 equiv. of doxorubicin could be homogeneously incorporated in the gels (Supplementary Fig. S3). For example, a hydrogel would form by adding 0.5 equiv. of doxorubicin and it was stable at room temperature for at least three months.

Rheology was then performed to characterize the mechanical properties of resulting gels with different amounts of doxorubicin (Fig. 2D and Supplementary Fig. S4). As shown in Fig. 2D, the results indicated that the G' (elasticity and storage modulus) value was bigger when the amount of doxorubicin was higher, and the G' value was about 10, 60, and 80 Pa for gels with 0.1, 0.2, and 0.3 equiv. of doxorubicin, respectively. For the gel with 0.1 equiv. of doxorubicin, the G' value increased a little bit when the frequency value was bigger than 10 rad/s, suggesting a shear thickening effect and a mechanical weak gel. For gels with bigger amounts of doxorubicin, their G' values were almost constant in the frequency range from 0.1 to 100 rad/s. The G' value of all gels was about 7 times bigger than their corresponding G″ value, indicating the formation of hydrogels.

### Cross-linking of the nanofibers by doxorubicin

**Figure 3:**
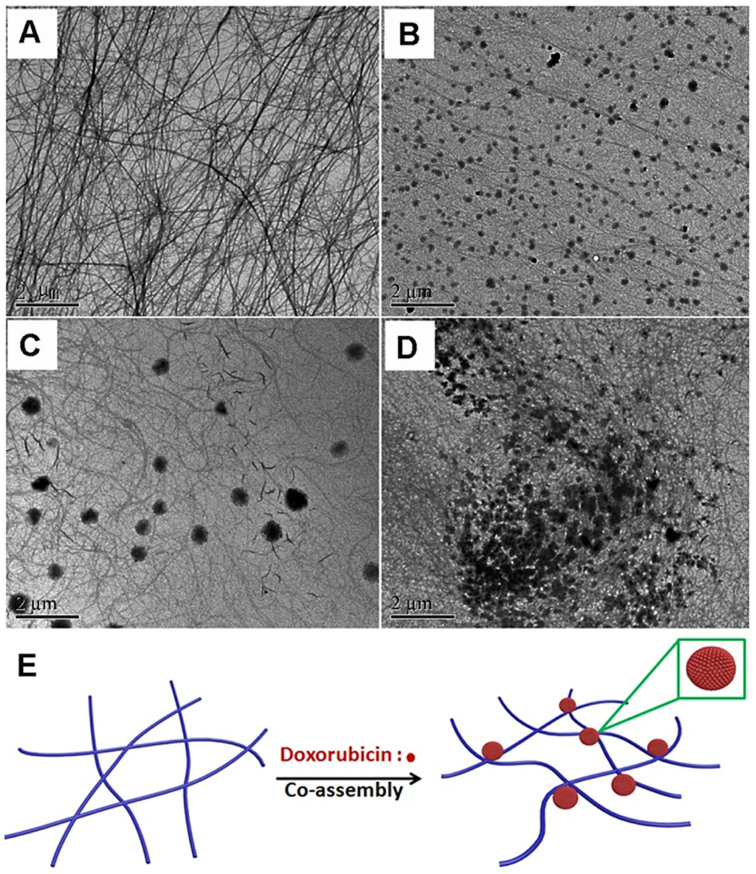
Cross-linking of the nanofibers by doxorubicin. TEM images of (A) the peptide in PBS solution at the concentration of 0.5 wt% and pH value of 7.4 and the gels formed by adding different equiv. of doxorubicin to the peptide solution: (B) 0.1 equiv., (C) 0.2 equiv., and (D) 0.3 equiv. as well as (E) an illustration for hydrogel formation by adding doxorubicin to the peptide solution.

**Figure 4:**
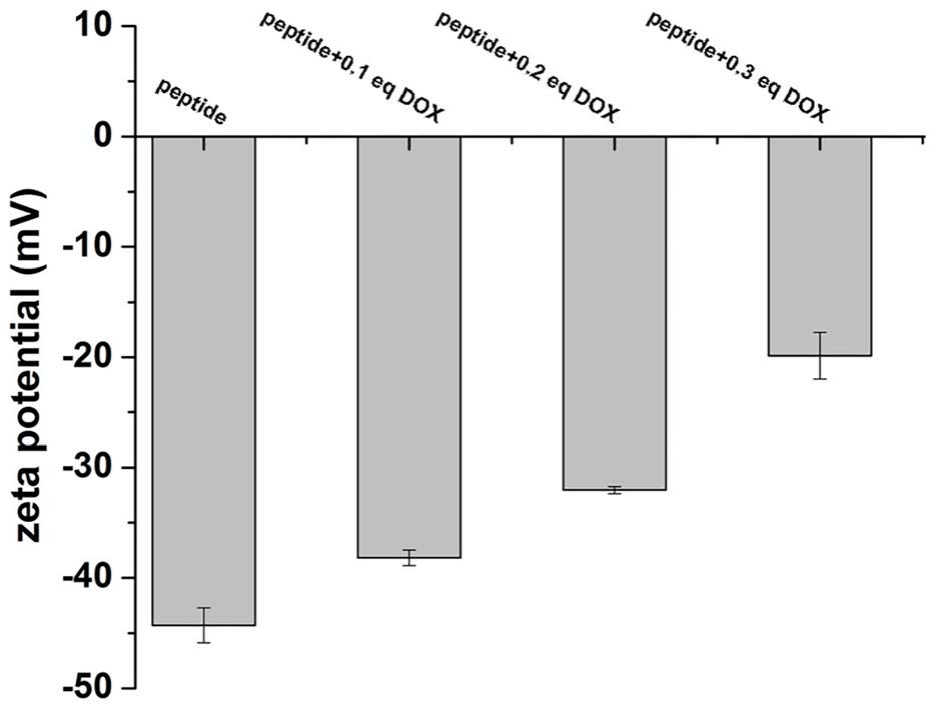
Zeta potential study. Zeta potentials of peptide solution in the absence of doxorubicin and upon addition of various equivalents of doxorubicins. DOX: doxorubicin.

Transmission electron microscopy (TEM) was then employed to characterize the morphology of self-assembled nanostructures in the peptide solution and the gels. As shown in Fig. 3A, the peptide self-assembled into uniform nanofibers with the diameter of about 40–50 nm in the PBS solution (0.5 wt%), which was also similar to Liu's reports^47^. For the gel with 0.1 equiv. of doxorubicin, we found nanospheres with the diameter of about 150 nm at the surface of nanofibers and the diameter of the nanofibers changed to be smaller (20–30 nm) (Fig. 3B). For gels with more amounts of doxorubicin, the diameter of nanospheres was larger and the diameter of peptide nanofibers remained similar to that in Fig. 3B, suggesting that the nanospheres were probably formed by doxorubicin (Fig. 3C). These observations were similar to the previous report that Janus nanogels could be formed by the phase separation between taxol and the PLGA-PEG-PLGA copolymer^48^. However, we could not totally rule out the possibility that the formation of nanospheres at the surface of nanofibers was due to the formation of peptide-DOX complex. In the presence of 0.3 equiv. of doxorubicin, the nanospheres were irregular and similar to precipitation (Fig. 3D). Stupp and co-workers have demonstrated that nanofibers of peptide amphiphiles are promising carriers for hydrophobic therapeutic agent of HCPT and the HCPT could be homogeneously encapsulated in the hydrophobic domain of nanofibers^43^. Liu's group also demonstrated that the peptide nanofibers of Nap-GFFYGRGD could homogeneously encapsulated HCPT for its delivery^47^. Our observations were different with their results and the hydrophobic doxurubicin could not be encapsulated in the nanofibers. Therefore, the doxorubicin grew into nanospheres at the surface of the nanofibers. Furthermore, the zeta potential study of peptide solution upon addition of various equiv. of doxorubicin at pH 7.4 was performed, as depicted in Fig. 4. The results indicated that the zeta potential of the peptide solution in the absence of doxorubicin was about −44 mV, which was significantly neutralized with the increase of doxorubicin amount in the peptide solution.

Based on these observations, we proposed a possible illustration for hydrogelations in our system (Fig. 3E). Similar to Liu's observations, the peptide itself could only form nanofibers but not hydrogels due to the relative weak inter-fiber interactions. The nanofiber was negatively charged and doxorubicin was positively charged. The addition of doxorubicin to the nanofiber solution led to the growth of nanospheres at the surface of nanofibers. The nanospheres then served as the cross-linkers to increase the inter-fiber interactions, leading to the formation of stable 3D fiber networks and hydrogels. Recently, Yang's group has reported on the using of tetrameric recombinant proteins to increase interaction of peptide nanofibers to form hydrogels for biomedical applications^49^,^50^,^51^. Our results suggested that nanospheres could also serve as the cross-linkers to increase inter-fiber interactions for hydrogel formation.

### Controlled drug release and cytotoxicity of the hydrogels

**Figure 5:**
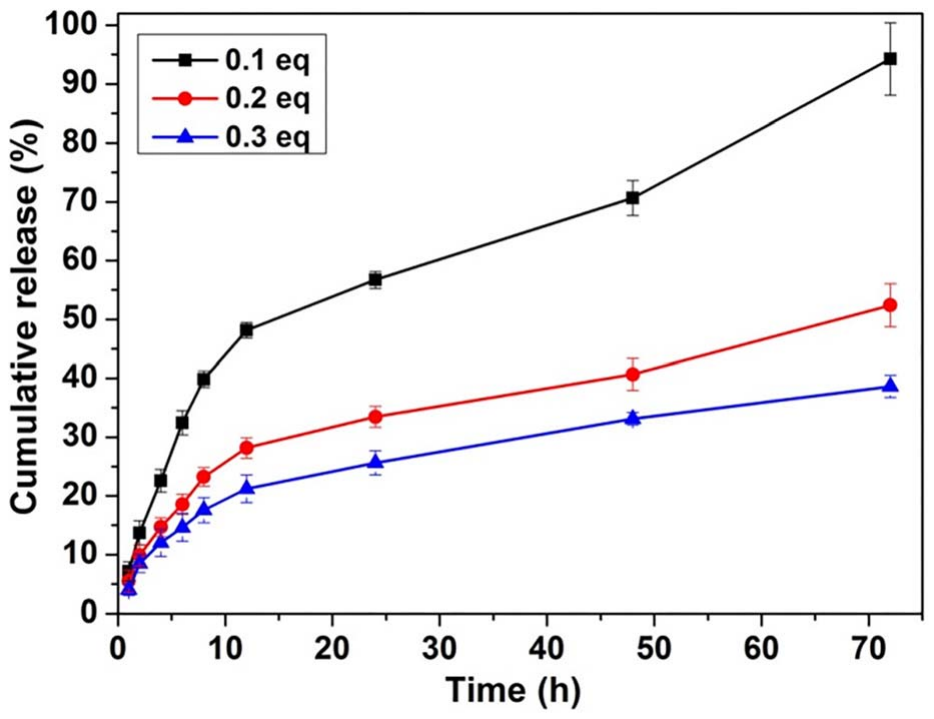
Controlled doxorubicin release from the hydrogels. Release profile of doxorubicin from gels with different equivalents of doxorubicin (the peptide concentration in PBS was fixed to be 0.5 wt%).

**Figure 6:**
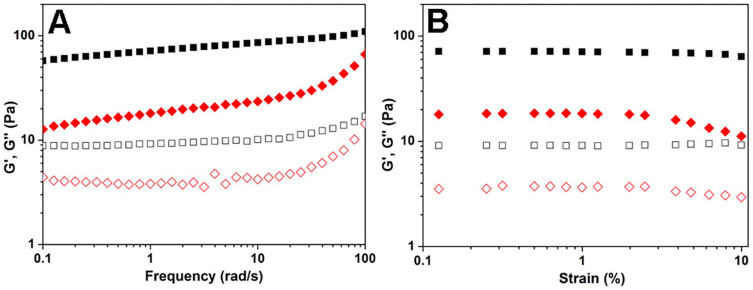
Mechanical property of the gels after drug release. Rheological measurement with the modes of (A) dynamic frequency sweep at the strain of 0.5% and (B) strain sweep at the frequence at 1 rad/s of the gels formed by adding 0.2 equiv. of doxorubicin to the peptide solution (squares: before doxorubicin release, diamonds: after doxorubicin release, filled symbols: G' and open symbols: G″).

**Figure 7:**
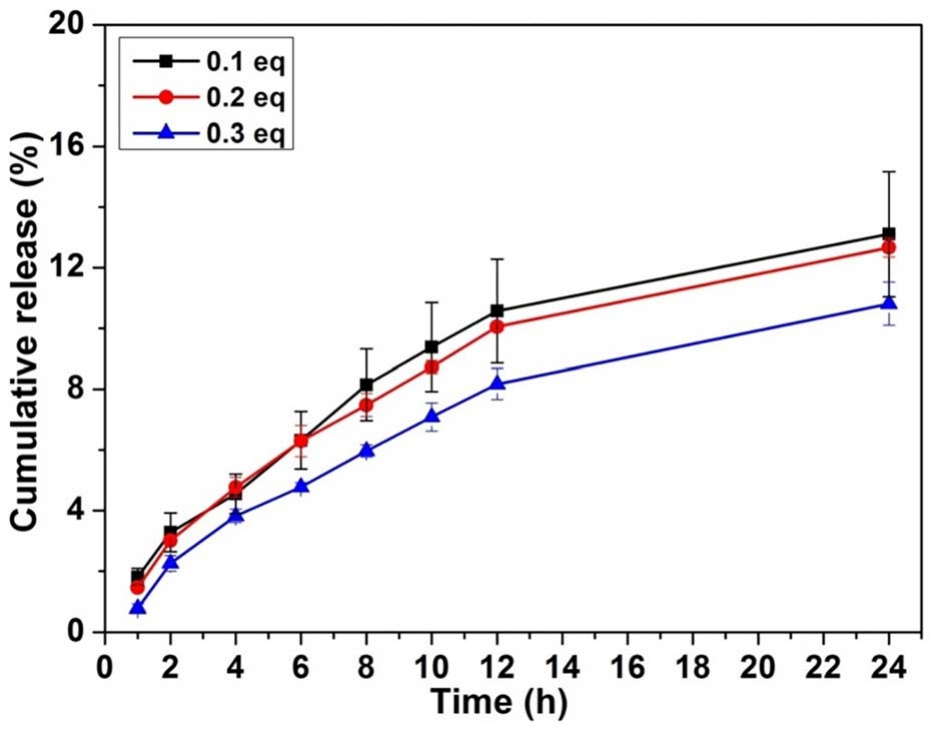
Peptide release from the hydrogels. Release profile of peptide of Nap-GFFYGRGD from gels with different equivalents of doxorubicin.

We then studied the release behaviour of doxorubicin from the gels at 37°C. A 250 μL of PBS solution was added to a 200 μL formed gel with different amounts of doxorubicin. A 200 μL upper solution was taken out at desired intervals and a fresh solution was added back to the system. The accumulated amounts of doxorubicin released from the gels were determined by measuring the absorbance of doxorubicin at wavelength of 580 nm. Fig. 5 showed the results, and higher percentages of doxorubicin got released from gels with less amounts of doxorubicin, suggesting that doxorubicin would release more rapidly from mechanically weak gels. During the 72 h experimental period, there were about 95%, 52% and 38% of doxorubicin released from gels with 0.1, 0.2, and 0.3 equiv. of doxorubicin, respectively. Furthermore, we have performed the rheological measurement of the gels (with 0.2 equiv. of doxorubicin) before and after drug release for 24 h. As shown in Fig. 6, the mechanical property of the gels became weaker after doxorubicin release. However, post 24 drug release, the G′ value remained to dominate G″ value, suggesting that the gels would not change to a sol but were still a viscoelastic hydrogel. As the gels were getting weaker, we next investigated whether the peptide would be released from the gels. Fig. 7 exhibited the results, which revealed that the peptide release profiles from the gels formed by 0.1, 0.2, and 0.3 equiv. of doxorubicin were similar. There were around 12% of the total peptides released from the gels at 24 h. After release, the diameter of both nanospheres and nanofibers changed to be smaller (Supplementary Fig. S5), suggesting that both the peptide and DOX got released from the gel.

**Figure 8:**
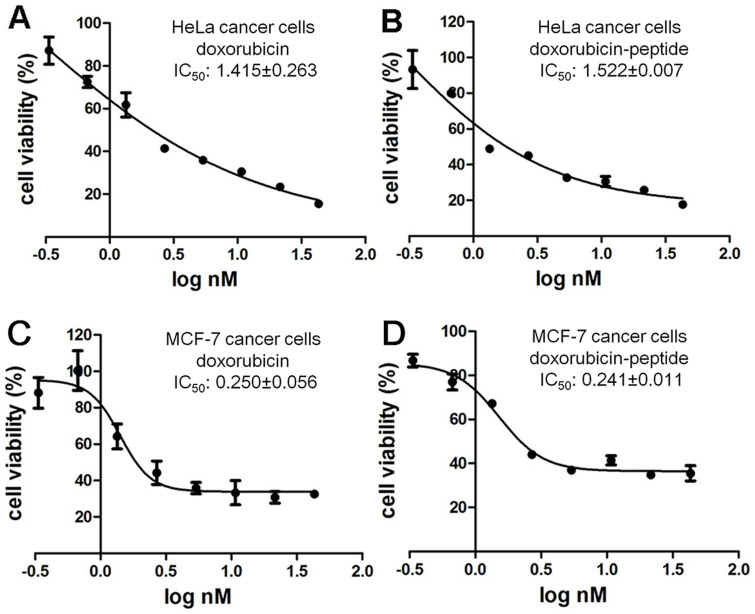
Cytotoxicity of doxorubicin-peptide hydrogels. Inhibition curves of (A) doxorubicin and (B) doxorubicin-peptide for HeLa cancer cells. Inhibition curves of (C) doxorubicin and D) doxorubicin-peptide for MCF-7 cancer cells.

MTT cell viability test was used to determine the anticancer cells activities. As shown in Fig. 8, the IC_50_ values of free doxorubicin and doxorubicin in nanofibers were similar for a variety of cancer cell lines such as HeLa and MCF-7 cancer cells, suggesting that such formulation would not decrease the anticancer activity of doxorubicin. Though the anti-tumor activity of doxorubicin was not improved by the hydrogel formulation as compared to free doxorubicin, supramolecular hydrogels might be applied for local and sustained release of doxorubicin to treat cancers and minimize the drug adverse effects. These observations suggested that our hydrogels might be applied for controlled delivery of doxorubicin.

## Discussion

We demonstrated that the supramolecular nanofibers of Nap-GFFYGRGD could not encapsulate doxorubicin, and therefore the incorporation of doxorubicin to the peptide solution led to the formation of nanospheres of doxorubicin at the surface of peptide nanofibers. The nanospheres could serve as cross-linkers to increase the inter-fiber interactions, resulting in the formation of stable 3D fiber networks and hydrogels. Our study provides a new strategy of using nanospheres as cross-linkers to form supramolecular hydrogels. Our hydrogels have great potential for the controlled delivery of anticancer drugs.

## Methods

### Chemicals

Fmoc-amino acids were obtained from GL Biochem (Shanghai). Doxorubicin hydrochloride was purchased from Aladdin. Commercially available reagents were used without further purification, unless noted otherwise. Nanopure water was used for all experiments. All other chemicals were reagent grade or better.

### General methods

The synthesized compounds were characterized using ^1^H NMR (Bruker ARX 400). LC-MS spectrometric analyses were performed at the LCMS-20AD (Shimadzu) system. HPLC was conducted at LUMTECH HPLC (Germany) system using a C18 RP column with MeOH (0.05% of TFA) and water (0.05% of TFA) as the eluents. Rheology was performed on an AR 2000ex (TA instrument) system using a parallel plates (40 mm) at the gap of 500 μm. MTT data was recorded on a BioTek Synergy™ 4 Hybrid Microplate Reader. TEM images were done on a Tecnai G2 F20 system, operating at 200 kV. The zeta potentials of the self-assembled nanofibers were measured by a zeta potential analyzer (Zeta Pals, Brookhaven Instruments, Huntsville, NY, USA). Before measurements, the hydrogels formed by adding different equiv. of doxorubicin to the 0.5 wt% peptide solution and peptide solution itself were all diluted for 5 times by 1 × PBS, affording corresponding nanofiber solutions. The zeta potential was then measured with palladium electrodes at 25°C, and the mean value of three readings was taken.

### Peptide systhesis

The peptide derivative was synthesized by solid phase peptide synthesis (SPPS) using 2-chlorotrityl chloride resin, the corresponding N-Fmoc protected amino acids with side chains properly protected by different group. The first amino acid was loaded on the resin at the C-terminal with the loading efficiency about 1.2 mmol/g. 20% piperidine in anhydrous N,N′-dimethylformamide (DMF) was used during deprotection of Fmoc group. Then the next Fmoc-protected amino acid was coupled to the free amino group using O-(Benzotriazol-1-yl)-N,N,N′,N′-tetramethyluroniumhexafluorophosphate (HBTU) as the coupling reagent. The growth of the peptide chain was according to the established Fmoc SPPS protocol. At the final step, 2-Naphthalene acetic acid was used to attach on the peptide. After the last coupling step, excessive reagents were removed by a single DMF wash for 5 times, followed by five steps of washing using DCM. The peptide derivative was cleaved using 95% of trifluoroacetic acid with 2.5% of TIS and 2.5% of H_2_O for 30 minutes. 20 mL per gram of resin of ice-cold diethylether was then added to cleavage reagent. The resulting precipitate was filtrated and washed by ice-cold diethylether. The crude product was purified by HPLC and dried by lyophilizer.

### Compound Nap-GFFYGRGD

^1^H NMR (400 MHz, DMSO) δ 9.19 (s, 1H), 8.29−8.17 (m, 4H), 8.13 (d, *J* = 6.8 Hz, 2H), 8.09−8.02 (m, 2H), 7.89−7.79 (m, 3H), 7.75 (s, 1H), 7.69 (s, 1H), 7.48 (dd, *J* = 8.9, 5.8 Hz, 2H), 7.42 (d, *J* = 8.6 Hz, 2H), 7.25−7.10 (m, 12H), 7.06 (d, *J* = 8.3 Hz, 2H), 6.66 (d, *J* = 8.3 Hz, 2H), 4.57−4.41 (m, 4H), 4.31 (d, *J* = 5.8 Hz, 1H), 3.74 (dd, *J* = 16.3, 10.9 Hz, 5H), 3.65−3.54 (m, 3H), 3.10 (d, *J* = 5.7 Hz, 2H), 2.94 (ddd, *J* = 17.6, 16.4, 6.3 Hz, 3H), 2.83−2.73 (m, 2H), 2.72−2.64 (m, 2H), 2.60 (dd, *J* = 16.7, 6.6 Hz, 1H), 1.80−1.67 (m, 1H), 1.52 (dd, *J* = 13.7, 8.5 Hz, 3H) calc. M = 1085.96, obsvd. (M + H)^+^ = 1086.70

### Formation of gels

Compound of Nap-GFFYGRGD was firstly dissolved in 1 × PBS with 2 equiv. Na_2_CO_3_ adjusting the pH to 7.4. Doxorubicin was dissolved in the DMSO at the concentration of 100 mg/mL as a stock solution. Subsequently, different equiv. of doxorubicin in DMSO was added to the peptide solution. A homogeneous mixed solution was obtained by heating and hydrogels was formed within 5 minutes. The final DMSO concentration in the hydrogel was lower than 1%.

### Rheology

The rheology test was done on an AR 2000ex (TA Instrument) system, 40 mm parallel plates was used during the experiment at the gap of 500 μm. The gels were firstly characterized by the mode of time sweep, followed by a dynamic frequency sweep in the region of 0.1–100 rad s^−1^ at the strain of 0.5% and a dynamic strain sweep at the region of 0.1%–10%.

### Preparation of TEM samples of compounds

A carbon-coated copper grid was vertically dipped into the hydrogel or solution for 5 seconds, washed by water twice and then placed in a desicator overnight before the TEM measurement.

### Drug release

Hydrogels in PBS solution containing 0.5 wt% of compound and different equiv. of doxorubicin (0.1 eq–0.3 eq) were formed in an Eppendorf tube at 25°C. After 24 h, we added 0.25 mL of PBS (containing 0.5%(v/v)Tween 80)on the surface of the hydrogels, 0.2 mL of solution was taken out at the desired time point and 0.2 mL of PBS was added back. For the following time points, 0.2 mL of PBS was taken out and 0.2 mL of PBS was added back at each point. We then monitored and calculated the release profile of doxorubicin from the gels by measuring the absorbance of doxorubicin at wavelength of 580 nm. The experiment was performed at 37°C in 3 parallel.

### Determination of IC_50_ values on cancer cells

The HeLa and MCF-7 cancer cells were seeded in a 96-well plate with the density of 10,000 cells per-well (total medium volume of 100 μL). 24 hours post seeding, the solutions with a serial of concentrations of doxorubicin-peptide mixture or free doxorubicin in 100 μL of medium were added to each well (five wells for each concentration). Cells without the treatment of the compounds were used as the control. The MTT assays were performed after an extra culture time of 24 hours. All compounds were removed and 90 μL of fresh medium was added for each well, 10 μL of MTT solution (5 mg/mL) was added and incubated for 4 hours in 37°C. Pipette out the spent media, formazon crystals at the bottom of each well were dissolved in 100 μL DMSO. After 15 minutes shaking at room temperature, absorbance at wavelength of 490 nm was tested using a BioTek Synergy™ 4 Hybrid Microplate Reader. The experiment was repeated for 3 times. IC_50_ values for the inhibition of cell viability were calculated from pharmacological inhibitory response curves using software Prism 5.0.

## Author Contributions

J.H. and D.D. designed the research. Q.X., H.R., C.X. and C.R. performed the experiments. Q.X., H.R., C.X., G.W., C.R., J.H. and D.D. analyzed data and participated in the discussion. J.H. and D.D. wrote and revised the paper. All authors reviewed the manuscript.

## Supplementary Material

Supplementary Information Supplementary Info
