# Supplementary material for: Nanospheres of doxorubicin as cross-linkers for a supramolecular hydrogelation
Source: Sci Rep. 2015 Mar 5;5:8764. doi: 10.1038/srep08764 (PMC4350081; doi:10.1038/srep08764)
Supplement: Supplementary Information — Supplementary Info [file srep08764-s1.pdf]

Supplementary Information

Nanosperes of doxorubicin as cross-linkers for a supramolecular hydrogelation

Qiang Xue[1]*, He Ren[1]*, Chao Xu[1], Gang Wang[2], Chunhua Ren[2], Jihui Hao[1] & Dan Ding[2]

[1]National Clinical Research Center for Cancer, Key Laboratory of Cancer Prevention and Therapy, Department of Pancreatic Cancer, Tianjin Medical University Cancer Institute and Hospital, Tianjin, P. R. China, [2]State Key Laboratory of Medicinal Chemical Biology, Key Laboratory of Bioactive Materials, Ministry of Education, College of Life Sciences, Nankai University, Tianjin, 300071, P. R. China.

Correspondence and requests for materials should be addressed to D.D. (dingd@nankai.edu.cn) or J.H. (haojihui@tjmuch.com). * These authors contributed equally to this work.

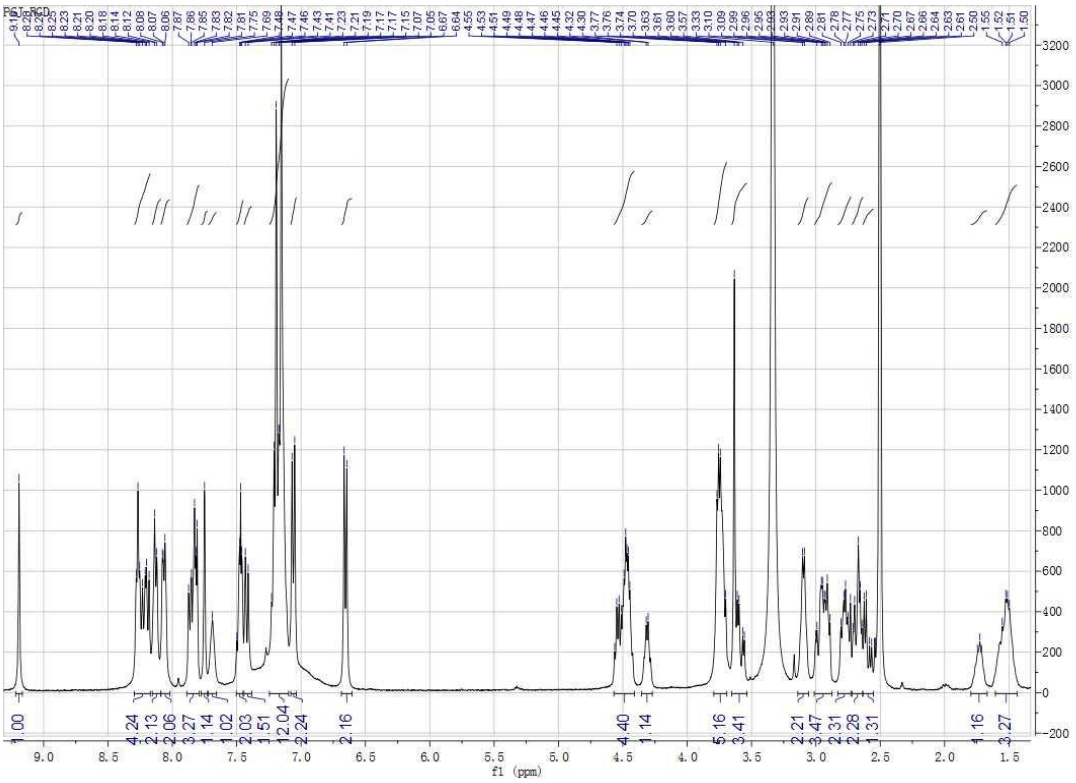

Supplementary Figure S1. ^{1}H NMR spectrum of the compound Nap-GFFYGRGD.

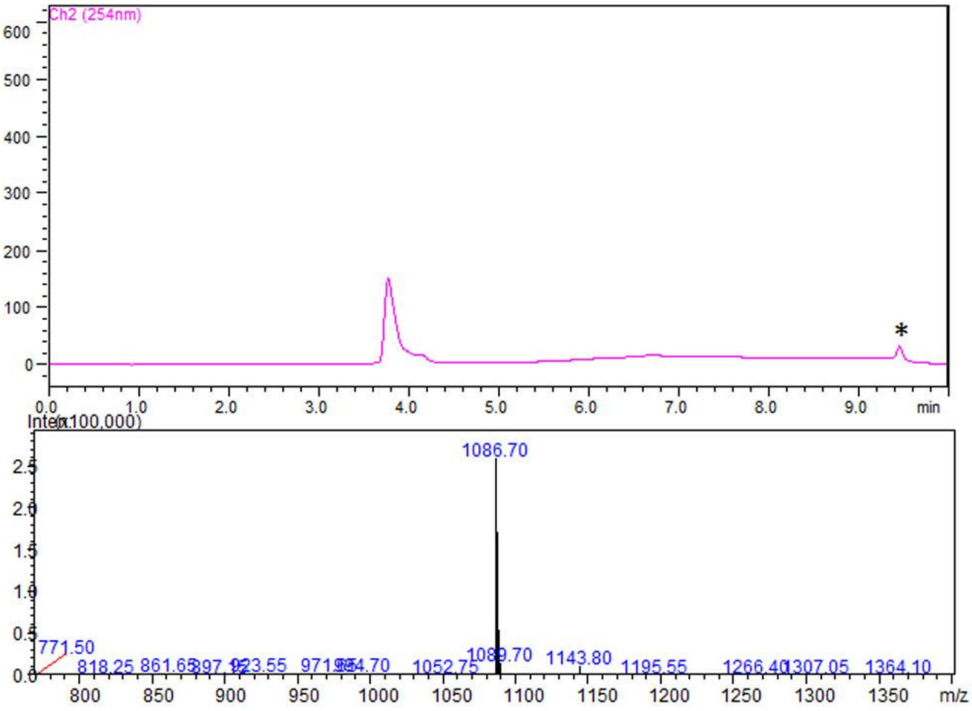

Supplementary Figure S2. LC-MS spectrum of the compound Nap-GFFYGRGD (peak marked with * is system peak).

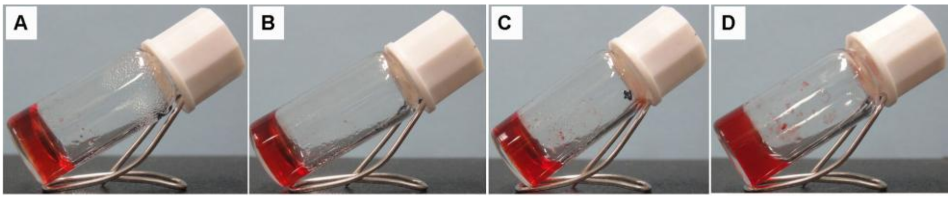

Supplementary Figure S3. Optical photos of hydrogels containing 0.5% peptide with A) 0.1, B) 0.2, C) 0.3 and D) 0.5 equiv.of doxorubicin.

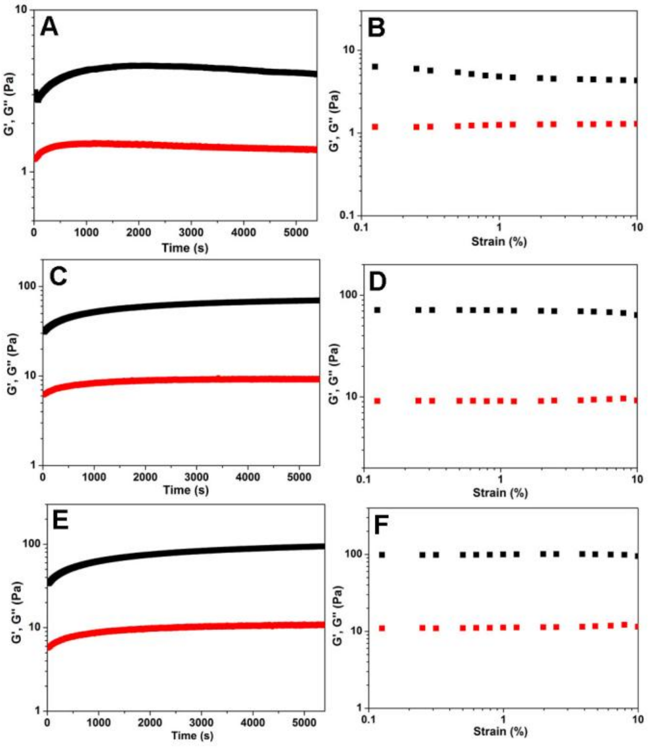

Supplementary Figure S4. Time sweep at the strain of 0.5% of hydrogels containing 0.5% peptide with A) 0.1, C) 0.2, E) 0.3 equiv.of doxorubicin and strain sweep of hydrogels containing 0.5% peptide with B) 0.1, D) 0.2, F) 0.3 equiv.of doxorubicin.

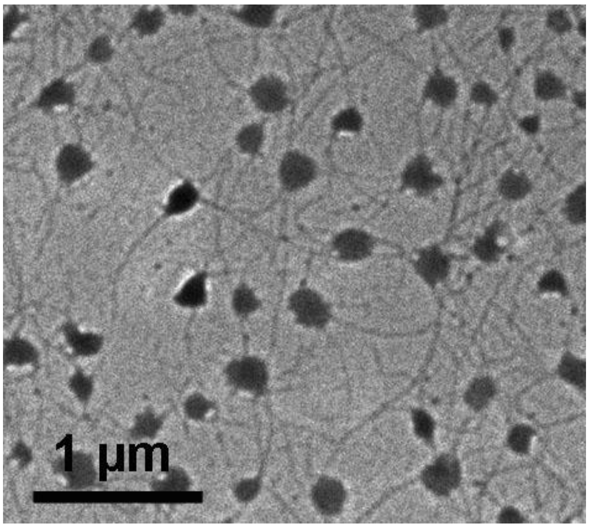

Supplementary Figure S5. TEM image of the gel with 0.2 equiv.of doxorubicin after release for 24 h.
